# First aid and treatment for cervical spinal cord injury with fracture and dislocation

**DOI:** 10.4103/0019-5413.36991

**Published:** 2007

**Authors:** W Yisheng, Z Fuying, W Limin, Li Junwei, P Guofu, W Weidong

**Affiliations:** Department of Orthopedics, the first Affiliated Hospital of Zhengzhou University, Henan Province, China

**Keywords:** Cervical spine, first aid, spinal cord injury, surgical treatment

## Abstract

**Background:**

Traumatic cervical spinal cord injury with subaxial fracture and dislocation not only indicates a highly unstable spine but can also induce life-threatening complications. This makes first aid critically important before any definitive operative procedure is undertaken. The present study analyzes the various first aid measures and operative procedures for such injury.

**Materials and Methods:**

Two hundred and ninety-five patients suffered from cervical spinal cord injury with fracture and dislocation. The average period between injury and admission was 4.5 days (range 5 h-12 weeks). The injury includes burst fractures (*n* = 90), compression fractures with herniated discs (*n* = 50), fractures and dislocation (*n* = 88) and pure dislocation (*n* = 36). Other injuries including developmental spinal canal stenosis and/or multi-segment spinal cord compression associated with trauma (*n* = 12), lamina fractures compressing the spinal cord (*n* = 6), ligament injuries (*n* = 7) and hematoma (*n* = 6) were observed in the present study. The injury level was C4 (*n* = 17), C5 (*n* = 29), C6 (*n* = 39), C7 (*n* = 35), C4-5 (*n* = 38), C5-6 (*n* = 58), C6-7 (*n* = 49), C4-6 (*n* = 16) and C5-7 (*n* = 14). According to the Frankel grading system, grade A was observed in 20 cases, grade B in 91, grade C in 124 and grade D in 60. One hundred and eighteen (40%) patients had a high fever and difficulty in breathing on presentation. First aid measures included early reduction and immobilization of the injured cervical spine, controlling the temperature, breathing support, and administration of high-dose methylprednisolone within eight hours of the injury (*n* = 12) and administration of dehydration and neurotrophy medicine. Oxygen support was given and tracheotomy was performed for patients with serious difficulty in breathing. Measures were taken to prevent bedsores and infections of the respiratory and urological systems. Two hundred and thirty six patients were treated with anterior decompression, 31 patients were treated by posterior approach surgery and combined anterior and posterior approach surgery was performed in a single sitting on 28 patients.

**Results:**

All patients were followed for 0.5-18 years (mean 11.8 years). At least one Frankel grade improvement was observed in 178 (60.3%) patients. In the anterior surgery group, the best results were observed in the cases with slight compressive fracture with disc herniation (44/50 patients, 88.0%). In the posterior surgery group, one Frankel grade improvement was observed in the cases with developmental spinal canal stenosis with trauma, lamina fractures, ligament injuries and hematoma (27/31, 87.1%). Most of the patients in the Frankel D group recovered normal neurological function after surgery. The majority of the patients with Frankel C neurological deficit (102/124) had the ability to walk postoperatively, while most of the seriously injured patients (Frankel A and B) had no improvement in their neurological function. Radiolographic fusion of the operated segments occurred in most patients within three months. Loss of intervertebral height and cervical physiological curvature was observed to varying degrees in 30.1% (71/236) of the cases in the anterior surgery group.

**Conclusion:**

First aid measures of early closed reduction or realignment and immobilization of the cervical spine, breathing support and high-dose methylprednisolone were most important in the treatment for traumatic spinal cord injury. Surgery should be performed as soon as the indications of spinal injury appear. The choice of the approach—anterior, posterior or both, should be based on the type of the injury and the surgeon's experience. Any complications should be actively prevented and treated.

Traumatic cervical spinal cord injury is a serious problem. Both respiratory and cardiovascular functions are compromised as a result of the neurological deficit.^1^ If not managed adequately in a timely manner, these conditions can be life-threatening. The injured cervical spine is usually highly unstable, which can cause further damage to the spinal cord if not securely immobilized prior to a definitive treatment.^1^,^2^ Thus, early closed reduction and immobilization of the injured cervical spine along with administration of high-dose methylprednisolone, dehydration and neurotrophy medicine are important first aid measures. However, controversy exists as to the optimal method for immobilization and the actual benefit from the administration of methylprednisolone.^1^,^3^ While a definitive operative procedure is needed for almost all patients with cervical spinal cord injury with fracture and dislocation, conflicting opinions remain over the appropriate approach of anterior or posterior surgery or both.^1^,^2^,^4^,^5^ By reviewing 295 cases with traumatic cervical spinal cord injury who had been treated surgically in the last 18 years, the present study evaluates the first aid measures and various operative procedures to evolve a sound protocol for managing patients with such injury.

## MATERIALS AND METHODS

Two hundred and ninety five patients, 196 males and 99 females with an average age of 30.5 years (range 18-78 years) with traumatic cervical spinal cord injury were included in the study from March 1988 to September 2006. The average period between injury and admission was 4.5 days (range 5 h-12 weeks). This period was within 48 h in 232 cases, 48 h to 1 week in 47 cases and over 1week in 16 cases. The various causes of injury included fall from a height (*n* = 152), motor vehicle accident (*n* = 122) and sports-related injury (*n* = 21). Injuries of the cervical spine included burst fractures in (n=90), compression fractures with herniated disc (n=50), fractures and dislocation (n=88) and pure dislocation (n=36). Other injuries observed in this study included developmental spinal canal stenoses and/or multi-segment spinal cord compression associated with trauma (*n* = 12), lamina fractures compressing the spinal cord (*n* = 6), ligament injuries (*n* = 7) and hematoma (*n* = 6). In these injuries, spinal cord injury was usually more extensive than the spinal injury as indicated by the high signal on the T2-weighted MRI. The distribution of the injury levels was C4 in 17 cases, C5 in 29, C6 in 39, C7 in 35, C4-5 in 38, C5-6 in 58, C6-7 in 49, C4-6 in 16 and C5-7 in 14. According to the Frankel grading system of spinal cord injury, grade A was observed in 20 cases, grade B in 91, grade C in 124 and grade D in 60. One hundred and eighteen (40%) patients had a high fever and difficulty in breathing on presentation.

Our first aid measures included keeping the airway unobstructed, monitoring vital signs and immobilization of the injured cervical spine at injury site and during transportation. Immobilization methods included the use of a rigid collar, halo-vest or traction with Gardner-Wells tong. The closed reduction of dislocation and realignment of the cervical spine was performed in an emergent fashion. Gardner-Wells tong traction was started at ten pounds, increasing it as required under radiographic control. For those cases sent to us within eight hours of the injury (*n* = 12), high-dose methylprednisolone was administered according to the second National Acute Spinal Cord Injury studies’ (NASCIS-2) protocol. Oxygen support was given and tracheotomy was performed for patients with serious difficulty in breathing. Measures were taken to prevent bedsores and infections of the respiratory and urological system. The patients were also administrated IV fluid, mannitol and nerve nutrition, ganglioside. The patients with history of serious spinal cord injury, cases with complex cervical fracture or dislocation or fracture and dislocation of over 48 h or more had high fever and breathing difficulty. They reported later after initial treatment at other hospitals by immobilization of the injured cervical spine and oxygen support or tracheotomy. These patients were treated by us continuously for the infections of the respiratory and urological systems and for bedsores and high fever. Closed reduction was attempted in patients with fracture and dislocation or pure dislocation (*n* = 124) and it failed in 25 patients. The patients were prepared for surgery as early as possible.

Standard right-sided anterior approach surgery was performed in 236 patients including those with burst fracture (*n* = 87), compression fracture with disc herniation (*n* = 50), fracture and dislocation (*n* = 79) and pure dislocation (*n* = 20). A subtotal vertebrectomy was performed for the burst fracture of the vertebrae. Discectomy was performed for slight compression with disc herniation. Resection of posterior longitudinal ligament was usually done for adequate spinal canal decompression. Bone defects were bridged with autogenous, tri-cortical, iliac bone with the addition of a plate fixation.

A standard single-door laminoplasty or laminectomy was performed as posterior surgery in 31 cases. These cases included multi-level developmental spinal canal stenoses and/or multi-segment spinal cord compression associated with trauma (*n* = 12), lamina fractures compressing the spinal cord (*n* = 6), ligament injuries (*n* = 7) and hematoma (*n* = 6). Combined anterior and posterior surgery at one stage was done in 28 cases. This group included those with spinal cord compression from both anterior and posterior sides (*n* = 12) and those with irreducible dislocation (*n* = 16).

## RESULTS

**Figure 1:**
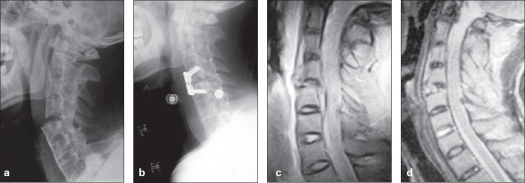
(a) X-ray of cervical spine (lateral view) of a 42 year-old male patient with ankylosing spondylitis shows C_4-5_ fracture and dislocation. (b) Postoperative X-ray of cervical spine showing anterior decompression, fusion and fixation with a plate, (c) Preoperative mid saggital T2WI of MRI shows cord compression, (d) Postoperative mid saggital T2WI of MRI shows the adequacy of reduction

**Figure 2:**
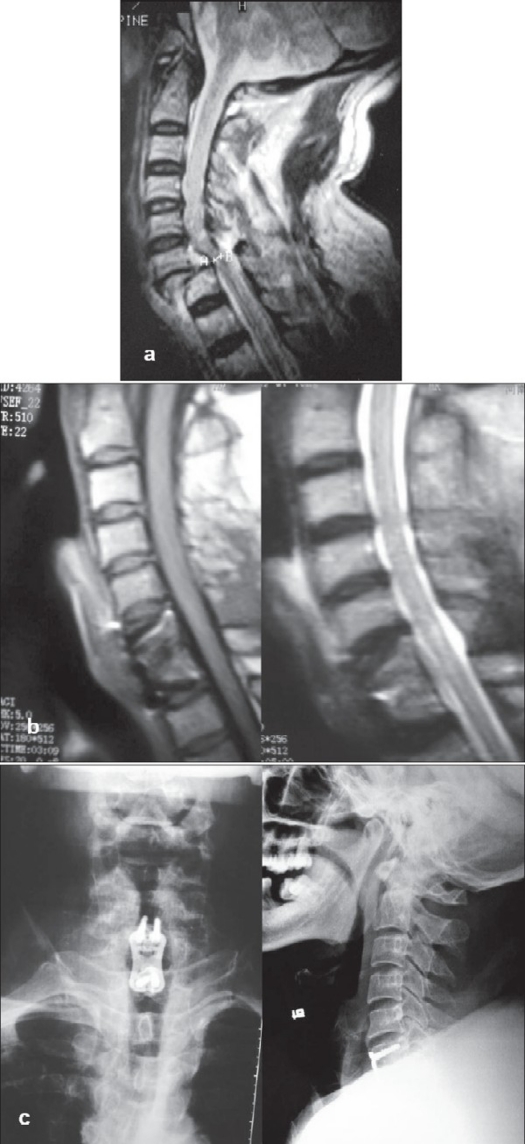
(a) T2WI of mid saggital MRI of cervical spine shows C_6-7_ fractures dislocation with cord compression. (b) T1WI and T2WI mid saggital MRI of the same patients shows reduction and adequate decompression of the cord. (c) X-ray cervical spine AP and Lateral of the same patient showing fusion and stabilization with plate after anterior decompression

All patients were followed for a mean period of 11.8 years (range 0.5-18 years). At least one Frankel grade improvement was observed in 178 patients (60.3%) [Table 1 and Figures 1, 2]. In the anterior surgery group, at least one Frankel grade improvement (to Frankel E or D) was observed in the cases with slight compressive fracture with the disc herniation (44/50 patients, 88.0%). For the posterior surgery group, at least one Frankel grade improvement (to Frankel E or D) was observed in cases with the indications described above including ligament injury and hematoma (87.1%, 27/31). 54 out of 60 patients in the Frankel D group recovered normal neurologic function after surgery. Most patients (102/124) in the Frankel C group with neurologic deficit had the ability to walk postoperatively, while 89/111 (80.2%) seriously injured patients (Frankel A and B) had no improvement in their neurologic function.

**Table 1:** Change in Frankel grade (pre- to postoperation)

		Postoperation				
		
Preoperation	*n*	A	B	C	D	E
A	20	18	2	0	0	0
B	91	2	69	20	0	0
C	124	0	2	20	102	0
D	60	0	0	0	6	54
E	0	0	0	0	0	0
Total	295	20	73	40	108	54

Radiolographic fusion of the operated segments was observed in the majority of the patients (241/264, 91.3%) within three months. At the final followup, loss of intervertebral height averaging 1.94 mm and loss of cervical physiological curvature averaging 8.35 degrees was observed in 30.1% (71/236) of the cases in the anterior surgery group. The loss of height in patients with anterior plate fixation was less than that in patients without anterior plate fixation. Fifteen per cent of the patients had recurrent myelopathy symptoms.

## DISCUSSIONS

Immobilization of the injured cervical spine is of vital importance.^1^,^2^,^6^ If this is ignored during transportation of the patient, the highly unstable injured spine can not only cause further damage to the spinal cord, but can also affect the respiratory function. Reduction or realignment of the cervical spine within the first few hours of injury may lead to dramatic improvement in the neurologic status. Reduction within two hours of injury may reverse tetraplegia.^6^ Early closed reduction can not only decompress the spinal canal indirectly but also decrease the need for more complicated surgical procedures. There is still disagreement as to the optimal method of immobilization. Sandbags or collars alone do not provide safe immobilization. However, it is safe and practical to apply the Halo-vest. Skull traction could be used when the halo-vest is not available.

In our department, emergency closed reduction is attempted routinely for alert patients with acute cervical spinal cord injury with dislocation. Gardner-Wells tong or Halo-vest traction was used. Once closed reduction or realignment of the injured segments was achieved by traction, the cervical spine was immobilized by maintaining tong traction plus a hard collar or a Halo-vest until a definitive surgery was performed. A Halo-vest was applied in the case of failed closed reduction (25 cases). There is no information available about the best method of immobilization while the patient is being transported to the operation room. In our experience, a hard collar or a tong traction may be inadequate unless the surgeon makes sure that the patient is safely transferred to the operation room. This is one important thing that should not be overlooked. In our experiences, the Halo-vest is the best option for such patients.

More attention should be paid to breathing and in case of serious difficulty in breathing, oxygen inhalation or tracheotomy should be performed as soon as possible. Temperature disturbances were observed frequently in spinal cord injury cases. Decreasing the temperature for patients with high fever and keeping the body warm in case of hypothermia are also important measures.

The minimization of the secondary spinal cord injury induced by the primary trauma is an important aspect for a patient with traumatic spinal cord injury. Lipid peroxidation and inflammatory response are important mechanisms of secondary cord injury. Glucocorticoid and dehydration medicines are effective for counteracting these mechanisms. The administration of neurologically protective drugs is also necessary.^3^,^6^ The efficacy of methylprednisolone in acute spinal cord injury has been questioned in recent years.^2^,^3^,^7^ The second National Acute Spinal Cord Injury studies' (NASCIS-2) protocol has been standard practice in many hospitals for the last decade. However, for patients with complete spinal cord injury, we have not observed any benefit from the use of high-dose methylprednisolone as compared to the cases from a decade ago when the second NASCIS protocol was not practised. Similar results have been shown by Tsutsumi *et al.*,^7^ who also proved the efficacy of the NASCIS-2 protocol for patients with incomplete paraplegia. Now, we use high-dose methylprednisolone only for patients with incomplete paraplegia within eight hours of injury.

The operation should be carried out as soon as possible if the operative indications exist. Preparation for surgery should be finished as early as possible. The timing of surgery for cervical spinal cord injuries remains controversial.^8^,^9^ Different outcomes have been reported as to early (<72 hours) *vs* late (>5 days) surgery for cervical spinal cord injury.^8^,^9^ In the present study, the patients underwent surgery as early as possible and we observed that a few cases had neurologic deterioration or increased complications. On the contrary, late surgery can be associated with more complications, which would make surgery less safe and necessitate the postponement of surgery until these complications were brought under control. These complications can be life-threatening and should be managed effectively so that surgery could be undertaken earlier to preserve uncompromised neurologic function.

The surgical approach can be chosen according to the source of cord compression, stability of the cervical spine and experience of the surgeon. Anterior, posterior or their combination can be chosen for a particular patient.^4^,^5^

Anterior compression of the spinal cord from disrupted vertebral body or intervertebral disc often indicates an anterior approach. The choice of a right-sided or left-sided approach depends on the surgeon's preference. In the present study, a left-sided approach was chosen only when fixation of T1 vertebra was needed (15 cases). We did not encounter any complications related to the approaches. When displaced fragments were removed for decompression, reconstruction was done in two ways. In the early years, we used Joggle-shaped iliac bone grafted technique, which securely implanted the grafting bone.^10^ In recent years, we prefer bone grafting and fixation with a plate for reconstruction.^11^,^12^ The longterm followup of the present study showed that intervertebral height and cervical physiological curvature were better maintained in most of the cases undergoing anterior fusion and plate fixation.

The posterior approach surgery can be used for removing the compression from the posterior side such as the lamina fractures, ligament injuries or hematoma. Patients with developmental cervical spinal canal stenoses or multi-segment spinal cord compression associated with trauma were also included in this group. In these injuries, the spinal cord injury was usually more extensive. Therefore, a more extensive decompression by a laminoplasty or laminectomy from C3 to C7 would provide a better environment for spinal cord recovery. In this group, at least one Frankel grade improvement (to Frankel E or D) was seen in 27/31 (87.1%) cases.

In cases where closed reduction of bilateral facet dislocation (16 cases) failed posterior open reduction followed by anterior fusion and plate fixation was performed. The anterior reduction and stabilization is another choice for an irreducible, locked facet joint. However, this technique requires specific instruments and is more demanding.^13^,^14^ The combined anterior and posterior surgeries greatly increase surgical trauma, blood loss and risk of complications. In our experience, adequate preoperative planning is very important in an attempt to shorten the operative time, decrease surgical trauma and blood loss. In the combined surgeries, the anterior or posterior procedures in themselves are not demanding, the key point is to make sure that the cervical spine be stable during the operation when the position of the patient has to be changed.

In the present study, the longterm outcome of partial spinal cord injury (SCI) cases showed improvement in neurological function, while no improvement was seen in the cases of complete SCI. The prognosis of SCI is reported to be poor.^2^,^3^,^6^,^8^ However, if the surgery for cord decompression and stabilization is indicated, the surgery should be carried out as soon as possible to provide an opportunity for neural recovery.

## CONCLUSION

The first aid measures of early closed reduction or realignment and immobilization of the cervical spine, breathing support and high-dose methylprednisolone are most important in the treatment of traumatic spinal cord injury. The surgery should be performed earlier in indicated cases. The choice of the approach-anterior, posterior or both, should be based on the type of injury and the surgeon's experience. The complications should be effectively prevented and treated.
